# Natural sampling and aliasing of marine geochemical signals

**DOI:** 10.1038/s41598-024-84871-6

**Published:** 2025-01-04

**Authors:** Andrew Curtis, Hugo Bloem, Rachel Wood, Fred Bowyer, Graham A. Shields, Ying Zhou, Mariana Yilales, Daniel Tetzlaff

**Affiliations:** 1 School of GeoSciences, University of Edinburgh, James Hutton Road, Edinburgh, EH9 3FE UK; 2 Department of Earth Sciences, University College London, Gower Street, London, WC1E 6BT UK; 3 Westchase Software, Houston, TX 77063 USA

**Keywords:** Biogeochemistry, Climate sciences, Environmental sciences, Ocean sciences, Solid Earth sciences

## Abstract

It is well known that the sedimentary rock record is both incomplete and biased by spatially highly variable rates of sedimentation. Without absolute age constraints of sufficient resolution, the temporal correlation of spatially disjunct records is therefore problematic and uncertain, but these effects have rarely been analysed quantitatively using signal processing methods. Here we use a computational process model to illustrate and analyse how spatial and temporal geochemical records can be biased by the inherent, heterogenous processes of marine sedimentation and preservation. This confirms that sedimentary hiatuses can span a substantial proportion of geological time, caused by inherent geological processes. Moreover, even in marine geochemical records that are essentially spatially continuous and complete, the signal is irreversibly disguised in time as lower frequency signals by an aliasing effect. We demonstrate that Nyquist’s theorem correctly predicts these biased signatures, proving that aliasing can be caused by cyclical and multiscale relative sea-level changes - a natural sampling effect. Our combined results show that deeper marine records are significantly more likely to provide unaliased environmental signatures. Also, some proxy residence times will be sufficiently long that they average over aliased frequencies, cancelling these in the geochemical record. And field observations of changes in sedimentation rate (such as hiatuses, condensation, or lateral expansion) can be used to infer possible aliasing. Where aliasing by natural sampling occurs, this cannot be undone simply by increasing sample resolution (density): aliasing is caused by an absence of sedimentary record, which by definition cannot be sampled at all. To overcome these issues, we propose that spatially separated aliased records may still be correctly correlated in age, and true geochemical cycles inferred, if a paired-sampling strategy informed by local stratigraphy is adopted. In this, two (or more) closely-spaced samples are analysed at each sampling point instead of only one, after which aliased cycles are inferred from geochemical gradients.

**Supplementary Information:**

The online version contains supplementary material available at 10.1038/s41598-024-84871-6.

## Introduction

“*The stratigraphic record is a lot of holes tied together with sediment.*”^1^

**Fig. 1:**
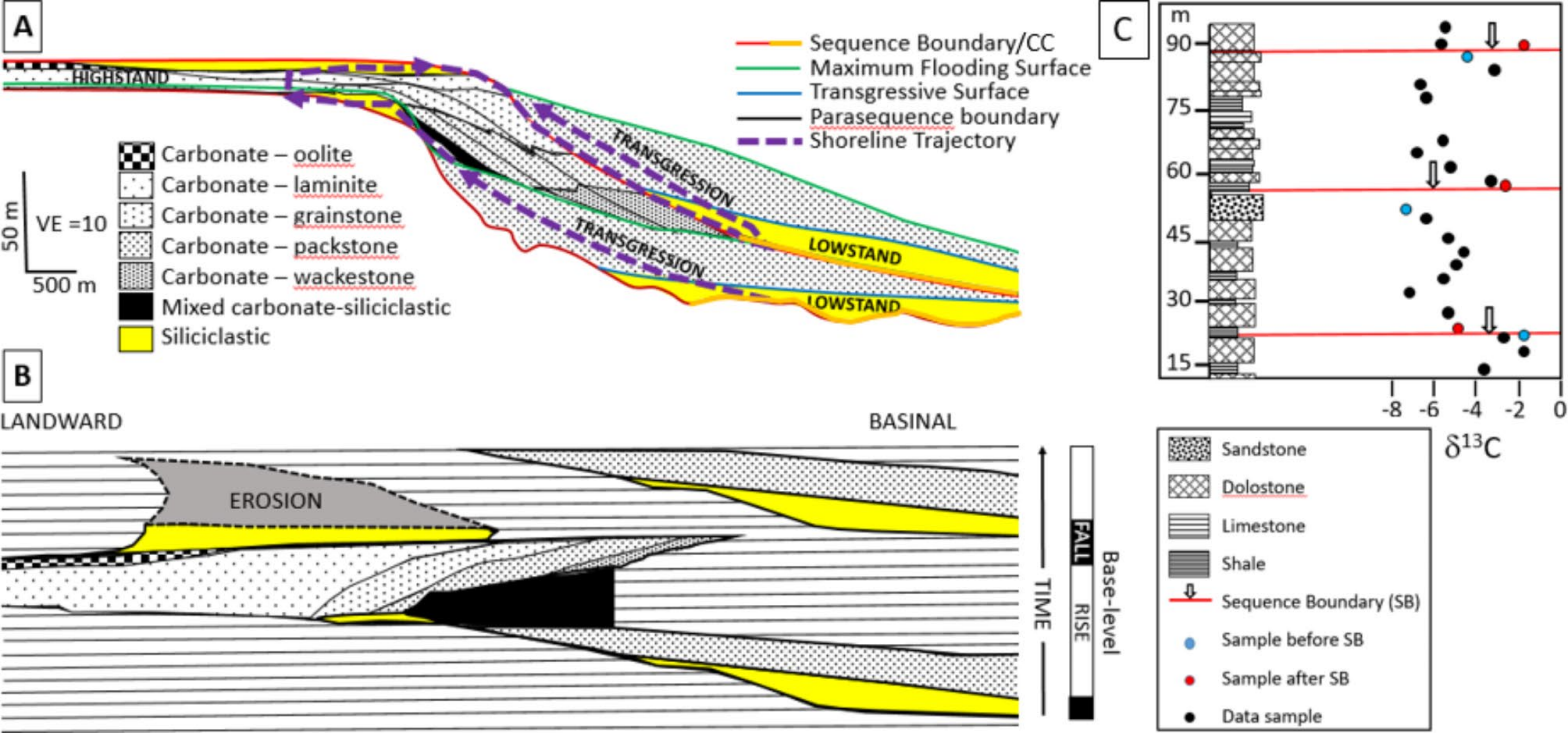
Mixed carbonate-siliciclastic sequence and δ^13^C data on a rimmed shelf. (**A**) Schematic, with facies distributions and major surfaces (sequence boundaries/correlative conformities (CC); transgressive surfaces; maximum flooding surfaces; parasequence boundaries) through a full and succeeding partial sequence. The shoreline trajectory traces the timeline of sediment at a fixed water depth through time. (**B**) A Wheeler diagram of (**A**) showing the actual distribution of facies in space (landward to seaward) and through time. Horizontal lines show non-deposition. Modified from^26^. VE is vertical exaggeration. (**C**) δ^13^C data from a vertical succession through a mixed carbonate-siliciclastic ramp succession at Brak, Ediacaran Nama Group (Omkyk Member), Namibia, showing the distribution of lithologies and the position of parasequence boundaries. Modified from^27^.

The incompleteness of the sedimentary record has long been established^1–3^. Stratigraphy is highly heterogenous in time and space, with sediments being absent, eroded, condensed or expanded, and successions are rife with long and obvious stratigraphic gaps (e.g., unconformities) as well as shorter, more cryptic ones (Fig. 1). Sediment accumulation rates are extremely variable, discontinuous and difficult to estimate, with rates appearing to decrease with increasing time span analysed, and the completeness of the record deteriorating over higher resolution time scales^3^. Indeed, numerical simulations show our knowledge and understanding of the sedimentary record to be biased by facies variations, hiatuses and unconformities, as well as incomplete experimental sampling^4–7^.

Accurate geochronological constraints are fundamental to our understanding of the evolution of the Earth, as they determine how a geological succession might be correlated both regionally and globally, enabling an accurate understanding of the temporal and spatial dynamics of secular change (e.g., compare^8,9^). By inverting age-depth modelling methods constrained by cyclostratigraphy – astronomically forced palaeoclimatic changes – high-resolution chemostratigraphic records can be understood. However, the incompleteness of stratigraphy and common absence of independent age controls remain unresolved issues that create ambiguity in interpretations, and obvious solutions are not yet evident^10^. Correlation strategies involve the estimation of sample ages between dateable strata by interpolation which is usually piecewise-linear. Depositional hiatuses are sometimes inferred in order to improve the correlation between data from disparate localities^11^. Such data are usually taken along an approximately bed-orthogonal axis assumed to follow a younging direction. Yet this methodology often leads to poorly constrained spatio-temporal sampling relationships because hiatus ages and their durations are unknown: if, for example, a hiatus occurs between each pair of consecutive samples then sample age estimates are almost arbitrary, constrained only by the law of superposition. Hiatus lengths can, however, be constrained through geological a priori information embodied within computational geological process models^48^ this method can improve correlations, estimate their Bayesian uncertainties, and create novel hypotheses (tomographic images of as-yet unmeasured geochemical signatures) with which correlations can be tested independently^12^. In all cases, improving the accuracy of the temporal interpretation of spatially distributed data reduces the risk either that true correlations are missed, or that spurious correlations are used to infer palaeoenvironmental change.

Many studies have shown that chemostratigraphic curves derived from marine sedimentary successions, most notably carbon isotope ratios (δ^13^C), can be incomplete, and globally-recognised excursions can be entirely absent locally^7,13,14^. The magnitude of δ^13^C variability can be far greater in deeper water records^15–18^, and many significant δ^13^C excursions coincide with a major rise in eustatic sea level^19^. Gradual geochemical shifts during such transgressive episodes may erroneously be interpreted as rapid events, as they are likely to be associated with decreased sedimentation rates. We would therefore predict an apparently abrupt initial change in the geochemical record (the ‘event’), followed by a more extended return (the ‘recovery’) to the long-term norm if sedimentation rates subsequently increase. Examples of this can be seen potentially at the Cenomanian-Turonian boundary, which in some sections is characterised by a relatively abrupt positive δ^13^C excursion^20^, but in others by a more symmetrical rise and fall^21^. Although we know that time is unlikely to be expressed linearly in a vertical section, this is still commonly assumed, leading to interpretations of unusually rapid or near instantaneous perturbation, e.g., in δ^44/40^Ca values^22,23^. In such cases, the recognition of astronomical cycles through expanded sections commonly reveals that rapidly changing δ^13^C values are expressed within an interval of anomalously thin cycles. In the case of the Cenomanian-Turonian event, the application of cyclostratigraphy shows that the carbon isotope recovery phase, far from being more gradual, was in reality more rapid than the “event” itself^21^.

These examples show that multi-site and perhaps spatially irregular sampling strategies are required to gain as complete a temporal record as possible. The geological record of each marine sedimentary basin is unique, so in turn each requires a bespoke sampling strategy. Sampling strategy is therefore key, but this begs the question, what are the limits to the record that can be observed?

While sedimentary forward models are commonly used to understand the completeness and dynamics of sedimentation, relatively few studies have sought to use sedimentary forward models to predict chemostratigraphic signatures within a sequence stratigraphic framework of changing accommodation space and sedimentation rate that incorporates different lithologies and unconformities. Myrow and Grotzinger (2000)^24^ used a forward model (STRATA) to show how marine stratigraphic processes (sediment flux and variable accommodation space based on sea level change) exert a fundamental control on hypothetical chemostratigraphic signatures (δ^13^C) across a shelf to basin profile. Within a single sequence, chemostratigraphic curves show gradual compression up-dip due to episodic condensation, and more abrupt compression basinwards due to reduced rates of sedimentation. Sedimentary records with lower mean accumulation rates therefore appear to record more rapid changes. In multiple sequences, δ^13^C values pre- and post-unconformities were increasingly offset up-dip, with proximal deposits being the least complete. In addition, these forward models show how sampling intervals can introduce further bias resulting in significant distortion, but suggest that dense (1 m) sampling of successions with very low sedimentation rates, and around unconformity surfaces, can potentially recover near complete records^24^.

Sequence stratigraphic analysis suggests that the spatial position at which any sedimentary facies is deposited migrates laterally during sea level fluctuations in a predictable way (Fig. 1A). The result is that a profile that samples depositional age uniformly in shallow marine sediments must usually migrate laterally as well as vertically^25^. In the resulting sedimentary sequence, the age of deposition therefore varies nonlinearly along any spatially-linear record that contains near-shore facies. Bed-orthogonal geochemical records typically cross unconformities where the age of deposition can change abruptly (Fig. 1B) and so cannot easily be constrained without careful analysis of surrounding sediments.

### Sampling and the interpretation of geochemical signatures

Knowledge of the water depth in which a marine sediment is deposited is critical to the interpretation of geochemical signatures, as the biological carbon pump leads to vertical depth gradients in isotopic ratios (e.g., of carbon, barium, cadmium), nutrient concentrations and redox conditions, and in the modern these are highly variable regionally^28^. Where significant, such gradients produce chemical disparities between contemporaneous but spatially distinct sites that are due only to different depths of deposition. Secular changes in geochemical signatures may then be confounded with depth gradients due to relative sea level changes in geochemical proxy data along individual bed-orthogonal records. Numerical simulations show that time is condensed near predictable sedimentary surfaces such as maximum flooding surfaces (MFS) and sequence boundaries (SB), driven by decreasing sedimentation, erosion and changes in water depth^6^. Increasing discrepancy in proxy values can therefore occur within the transgressive systems tract of the deep basin, in proximal upslope settings, and near the shelf break of the late highstand to early lowstand.

Sampling practice can also further limit our understanding of any given record. Often only a narrow range of lithologies such as carbonates are sampled (particularly in outcrop), ignoring other lithologies (e.g., shale) which may be poorly exposed, friable, and yet may represent the largest proportion of time in any given succession^7^. In some cases, samples from the same sequence but of different lithologies are studied separately so that any holistic sedimentological understanding is lost. Finally, carbonate rocks have highly variable proportions of depositional versus diagenetic, or neritic versus pelagic components^29^, and these proportions can vary between different geological settings and different intervals of geological history, thus complicating their utility as consistent environmental indicators.

These issues are illustrated in carbon isotope (δ^13^C) data from a ca. 80 m vertical record through a terminal Ediacaran mixed carbonate-clastic ramp succession^27^, in which we note several phenomena (Fig. 1C). First, there is a considerable offset, in this case up to ca. 3 to 4.5‰, in sample δ^13^C values found closely below and above the three traversed sequence boundaries. These are present as abrupt shifts to both more negative and more positive values, despite an expectation that changes would at least be consistent across cycle boundaries (Fig. 1A, B). Second, while there is considerable variability in δ^13^C values within any one sequence (up to ca. 4.5‰), these data generally show trends through time rather than random values (Fig. 1C). The variety of lithologies present within a sequence include shale, limestone, dolostone and sandstone, suggesting that these follow relative, cyclical sea level trends during the deposition of a sequence, potentially explaining the smoothness of δ^13^C trends. Finally, abrupt offsets are present, e.g., at ca. 80–85 m, which cannot readily be explained by the stratigraphic dynamics shown in Fig. 1. In sum, these observations indicate that there is far greater complexity in the interpretation of such vertical δ^13^C records than is normally appreciated, and a more sophisticated sequence model than that idealised in Fig. 1A is required.

### Conceptual model, and discussion of caveats, towards a new sampling strategy

While many of these sampling issues have long been recognized and idealized sampling strategies for stratigraphic sections with time-constrained intervals proposed. e.g., see review^7^, they have rarely been quantified or analysed using signal processing methods. Such methods can be used to analyse information transmitted and received in communication settings, and since geological preservation is a mode of communicating information about past environmental change with present observers, we show herein that even basic signal processing applied to present day signals may reveal novel information.

We first explore more quantitively the potential impact of variable sedimentation on hypothetical marine chemostratigraphic records. We then suggest stratigraphically controlled sampling strategies, and further probe the limits of the record – in part to identify that which we can never know. Finally, we show how a paired-sampling strategy combined with signal processing may reveal some of the apparently missing information.

We illustrate these concepts using a model-based simulation of stratigraphic sequences of an idealised mixed carbonate-clastic margin, both without, and with, an assumed major δ^13^C-depth gradient, and assigned 100 m sea-level oscillations (see Methods; SI; Fig. S1). This simple sea-level model scenario approximates to an icehouse period, e.g., the early Permian.

Our modelling approach is used to identify and illustrate potential emergent effects. The specific model results are in no way proposed to be universally applicable. Nevertheless, this conceptual framework allows us to interrogate important issues of incompleteness and bias. Our models assume that relative sea-level and water depth are major controls on sediment deposition. We recognise that the sea level scenarios chosen here are simple, and non-hierarchical, whereas the geological sea-level record is in fact composite, with many superimposed frequency cycles of sea-level change. Such complex cycles of accommodation space change will be reflected in part in the stratigraphy, and the effect of this in the fidelity and biases of any given record has been demonstrated in several more complex scenarios^4–6^. Each record will have a unique distribution of incompleteness, facies bias, and unconformity bias. The results derived here are therefore not intended to be widely applicable directly, but rather to be illustrative of a universal issue, and to demonstrate the utility and insight that can be gained from such a modelling approach.

More specifically, our model is used to explain specific processes and products, in order to understand how extrinsic factors such as relative sea-level changes (i.e., allogenic processes) can create a biased record in order to create testable hypotheses. Our models do not apply where autogenic processes (those intrinsic to the dynamics of sedimentation) are known to be dominant, as these can variably modify, obscure or completely remove any allogenic signal^30^. This includes potentially emergent autogenic behaviour, such as that of source to sink systems including turbiditic flow downslope that might be controlled mainly by complex underlying topographies through which sediments are directed and finally deposited in deep water fan settings. In such cases, the relatively importance of allogenic and autogenic controls may not always be easy to distinguish^30^.

We also assume that the modelled geochemical signature is primary and varies with water depth, can be corrected for diagenesis, and we make no assumptions as to the processes that create the signature. Systematic and highly variable carbonate δ^13^C values can occur in different, but time-equivalent, facies, which in turn are used to infer regional or global scale stratification of δ^13^C with water depth^31^. We recognise that some of the proximal-to-distal differences seen in δ^13^C records may be diagenetic in origin, or may rather be linked to different water masses rather than to water depth. Indeed, some δ^13^C records may also potentially correlate better with an onshore-offshore gradient rather than water depth itself, or are caused by differing fractions of transported (e.g., neritic versus pelagic) material and so would not reflect the isotopic composition of the water column. Nevertheless, such processes themselves may also have a depth-dependent control, as shown by Ediacaran examples^32,33^. So here we explore the effects of sampling as might be used to constrain this gradient, and test the effect of a δ^13^C water column gradient on the resultant modelled records. Although this may mean that different δ^13^C regimes might be recorded in some measured datasets, here for simplicity we assume a linear gradient.

**Fig. 2:**
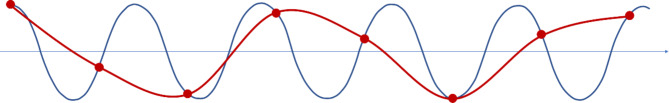
Illustration to show how the aliasing effect occurs if sampling is less dense than the Nyquist frequency (two samples per oscillation) of a true signal (blue). If samples are only recorded at the red dots, then the apparent frequency of the inferred signal (red curve) is lower than the true frequency.

The significant outcome is that we show how the intrinsic processes controlling shallow marine sedimentation can create datasets that can be irreversibly disguised by aliasing – a phenomenon in which rapid variations present at far lower temporal frequencies. We show that an aliasing effect occurs because temporal sampling can violate Nyquist’s theorem (Fig. 2), which states that more than two samples per oscillation (the Nyquist sampling frequency) are needed in order to record any oscillating signal unambiguously^34^. Whenever regular sampling is less dense than the Nyquist frequency, the true oscillation frequency is irreversibly expressed as a signal at a lower *apparent* frequency. We conclude that contemporaneous records above and below wave-base may often present ostensibly unrelated palaeoenvironmental signatures.

Finally, we propose a new paired-sampling methodology which may rectify current uncertainties. Aliasing might be detected in measured data using spatial derivatives – specifically, the sign of each gradient observable from closely spaced samples. This indicates that data can in fact include a higher frequency signal with peaks or troughs between each pair of sample points, revealing that aliasing has occurred. We therefore show with our real data set that aliasing can be detected, and that the correct cyclicity can in some cases be recovered using derivatives.

## Results

### Relationships between location, water depth, facies and depositional age

**Fig. 3:**
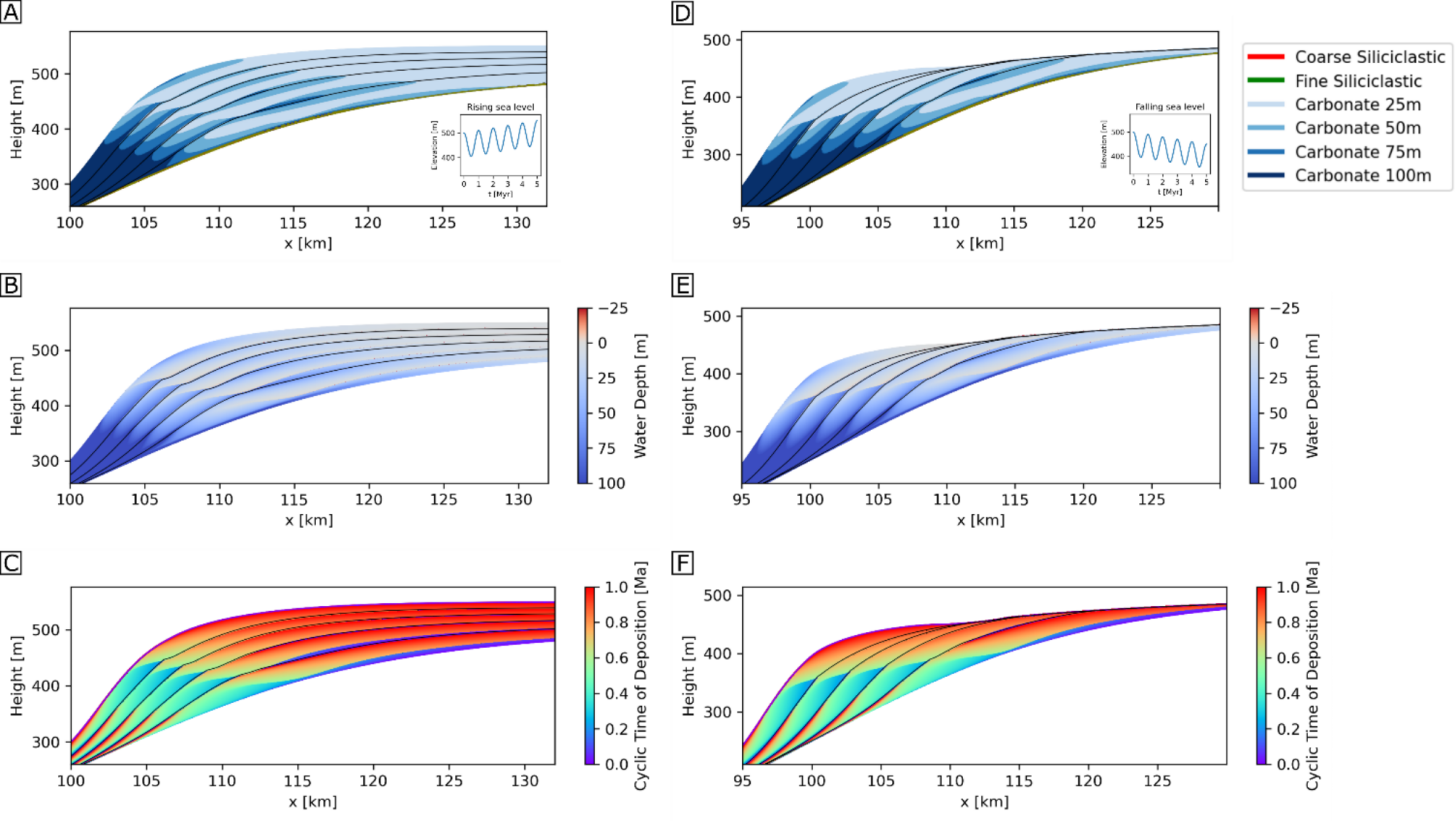
Section through 3D sedimentary succession simulated by geological process model SedSimple over 5 Myr with five 1 Myr relative sea level cycles.Overall rising sea-level (**A** inset) scenarios (**A-C**), and falling sea-level (**D** inset) scenarios (**D-F**) are shown. (**A,D**) Distribution of sedimentary facies. Facies colour is a mixture of individual facies colours in the legend, proportionate to their concentrations. Carbonate facies are discriminated based on the depth range of deposition (25 m: up to 25 m; 50 m: 25–50 m; 75 m: 50–75 m; 100 m: 75–100 m). (**B, E**) Water depth at time of deposition. (**C, F**) Deposition time within each 1 Myr cycle.

Landward-to-seaward cross sections through two sets of modelled sequences are shown for rising sea level (Fig. 3A-C) and falling sea level scenarios (Fig. 3D-F). For both, almost all of the rock volume on the slope comprises carbonate sediment (siliciclastics occupy only thin layers upon sequence boundaries), and the four carbonate facies are distributed in approximately similar patterns within each sequence (Fig. 3A, D). These facies distributions correspond to changing water depth in which each was deposited (Fig. 3B, E), and time of deposition within each 1 Myr cycle of the final rock volume (Fig. 3C, F).

**Fig. 4:**
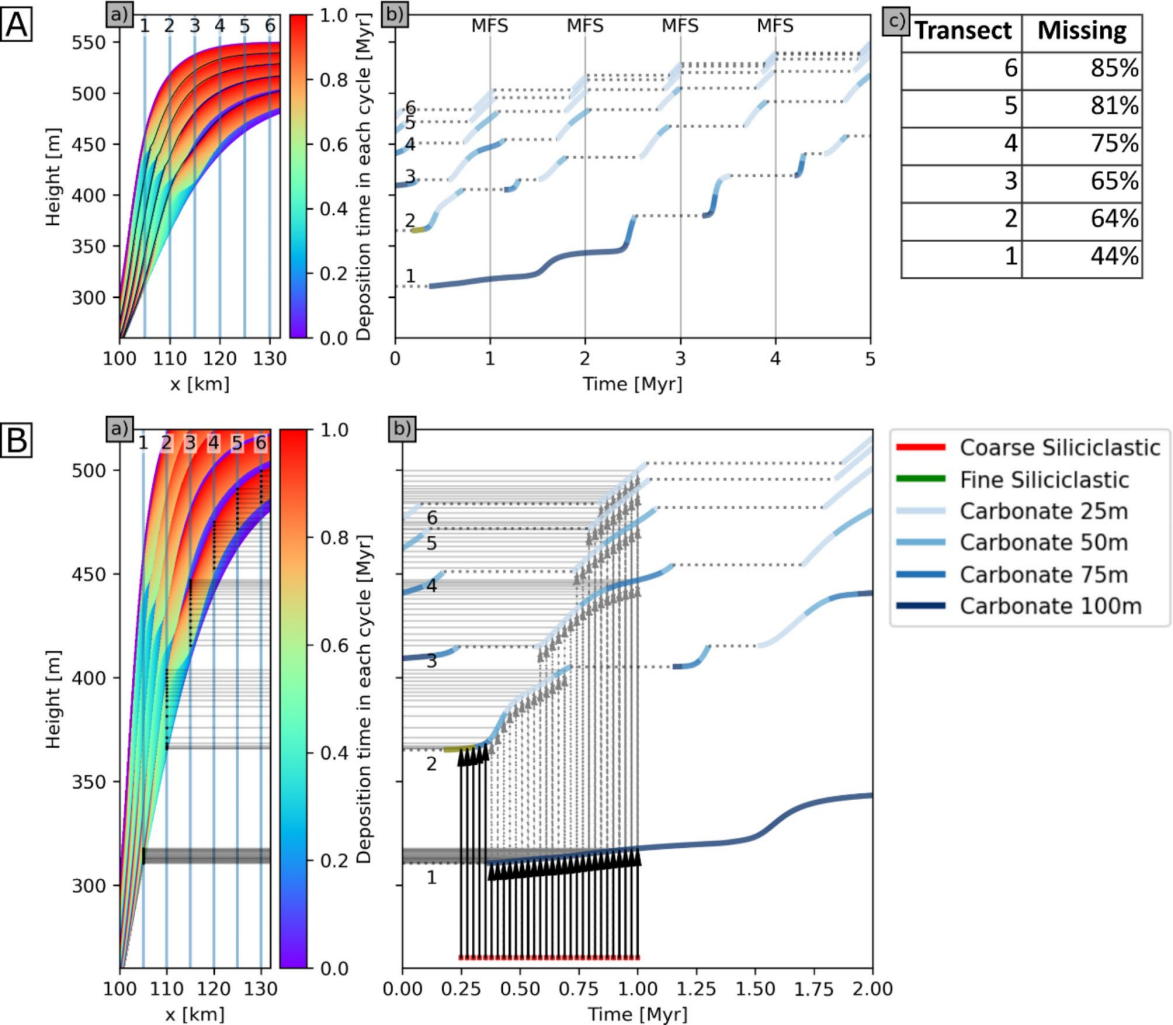
Cross-sections and vertical records through 3D sedimentary succession simulated by geological process model SedSimple with 1 Myr sea level cycles with an overall rising sea-level. (**A**) Location of six vertical records (1–6) over 5 Myr to show distribution of (a) timing of deposition. (b) facies deposited, with dotted horizontal sections of curves showing hiatuses. (c) total percentage of time missing (hiatuses) in each record. (**B**) Detail with (a) vertical exaggeration of records over first 2 Myr showing the distribution of sample locations on each vertical record (small triangles) that represent regular sampling of geological time . (b) facies deposited, with dotted horizontal sections of curves showing hiatuses. Regularly spaced geological time samples (red dots between 0.25 and 1 Ma) are projected (arrows) to elevations on each record at which those times are recorded in the sedimentary record. Dashed arrows show time samples preserved on more than one record.

From these models, the trajectories of elevation (vertical axis), depositional age (horizontal axis), and facies type (colours) of sediments preserved along six vertical records that are geographically distributed across a cross-section from the seaward, deeper basin (record 1) to the near shore (record 6) can be derived (Fig. 4, panels (b)). The distribution of intervals of hiatus on each record are shown as dotted horizontal sections on each curve. Different parts of the relative sea-level curve are sampled in the overall rising (Fig. 4).

compared to the falling sea-level scenarios (Fig. S2). Both scenarios sample the start of the sequence boundary formation in the first cycle, but this may be an artefact produced by the initiation of the model run. After this, in the overall rising sea-level scenario, samples lie dominantly in the transgressive systems tract in all cycles, but with temporal coverage decreasing, and terminating at the MFS in most records in later cycles. Most terminations correspond to sequence boundaries observed in Fig. 3, with the onset of each hiatus starting within the latest transgressive systems tracts. In shallower records these are approximately temporally coincident with the maximum flooding surface, and some extend into the highstand sequence tract.

In both simulations of overall rising and falling sea level, hiatuses span more than 50% of geological time on all but the seaward-most record, and increase to 86% and 95% for the most landward records in each simulation, respectively (Fig. 4(c); S2(c)). The overall length of time recorded in the sedimentary succession is greater in the falling sea-level scenario since increased erosion of shallow sediments results in more continuous deposition in basinward records, and in both scenarios the deepest records contain the most complete temporal records.

### Implications for the experimental design of geochemical sampling

Spatial samples that represent regular time intervals can be identified on each record. Regular points spanning the period 0.4 to 1 Myr (red dots, Fig. 4B(b)) are projected upward to intersect the set of depositional trajectories (vertical arrows). If non-zero gradients are encountered in any trajectory, this indicates that sediment deposited at that time is preserved in the corresponding record. Projecting such intersection points across to the elevation axis provides an exact location of the corresponding sample (horizontal arrows). Samples on other records may also record the same point in time (dashed arrows).

Regular samples of geological time are distributed unevenly in space on each record; by corollary, regularly-spaced sampling along any vertical record represents geological time irregularly (Fig. 4B(b)). The projected locations of those regular time samples represent an experimental design for spatial sampling along the set of records that records geological time without bias. Many but not all times can be observed on multiple lateral records, but importantly there is no time that can be observed in all records; obtaining a record that spans all times even within only this interval inevitably requires sampling in different geographical locations.

The abundance of hiatuses with significant and variable duration in vertical sampling records creates difficulties for the temporal interpretation of recorded data if environmental conditions and hence geochemical proxies vary temporally (secularly), laterally (spatially) or both. Geochemical gradients of δ^13^C, as well as redox gradients inferred from redox-sensitive trace element concentrations and isotopes in the water column, can only be constrained if signature variations of contemporaneously deposited samples from different water depths are available^33,35^. This inevitably requires samples from laterally offset locations identified by sequence stratigraphic analysis. If either the gradient or the overall environmental signature varies secularly, some intervals of time contain insufficient data to constrain the gradient, as only one, or even in some cases no, samples are preserved (Fig. 4B(b)).

**Fig. 5:**
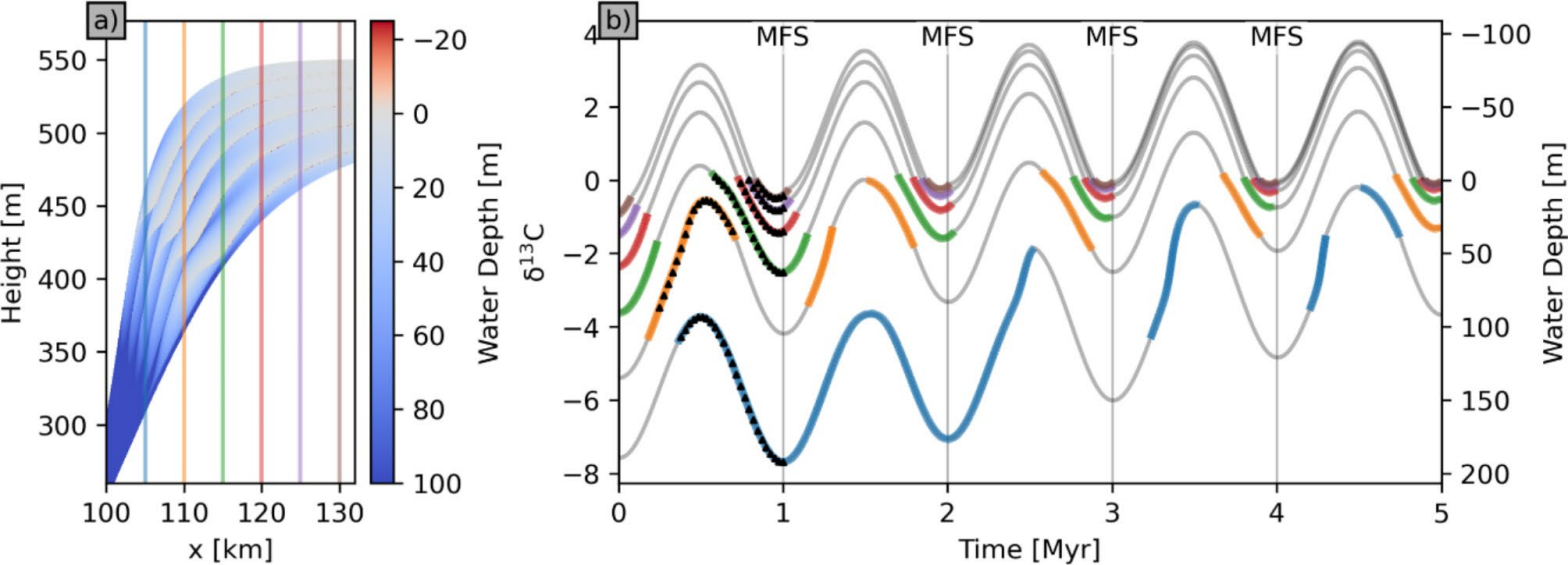
Sections through 3D sedimentary succession showing water depth changes simulated by geological process model SedSimple over 5 Myr with five 1 Myr sea level cycles during overall rising sea-level. (**a**) Water depth of deposition within each 1 Myr cycle, with locations of six vertical records (colour coded). (**b**) Water depth and δ^13^C values in each (colour coded) record through time, assuming gradient shown on left vertical axis. Grey lines show full water depth history along each record. Black dots show values at the regularly spaced time intervals between 0.25 and 1 shown in Fig. 4B(b). MFS = Maximum Flooding Surface.

Water depth at time of deposition varies significantly within, and between, different vertical records in both scenarios (Fig. 5). Using the example gradient of^36^, insertion of a δ^13^C axis creates significant scatter in the resultant δ^13^C values (Fig. 5B; S3B). If water depth is not known, data from samples deposited in different water depths are usually combined without correcting their measured signatures to a common water depth datum. This induces scatter that obscures the signature of true, secular environmental change (environmental conditions, represented by the chemical gradient in the water column, are temporally constant in these simulations).

If recorded seawater geochemical signatures change both with relative water depth (spatially, synchronously) due to vertical chemical gradients, and secularly (temporally) due to environmental dynamics, then these spatiotemporal effects may be confounded in recorded data. Here we further analyse results with these effects separated, revealing concomitant issues for regional and global correlation of geochemical signatures.

### Spatial geochemical gradients with respect to water depth, but no secular variation

**Fig. 6:**
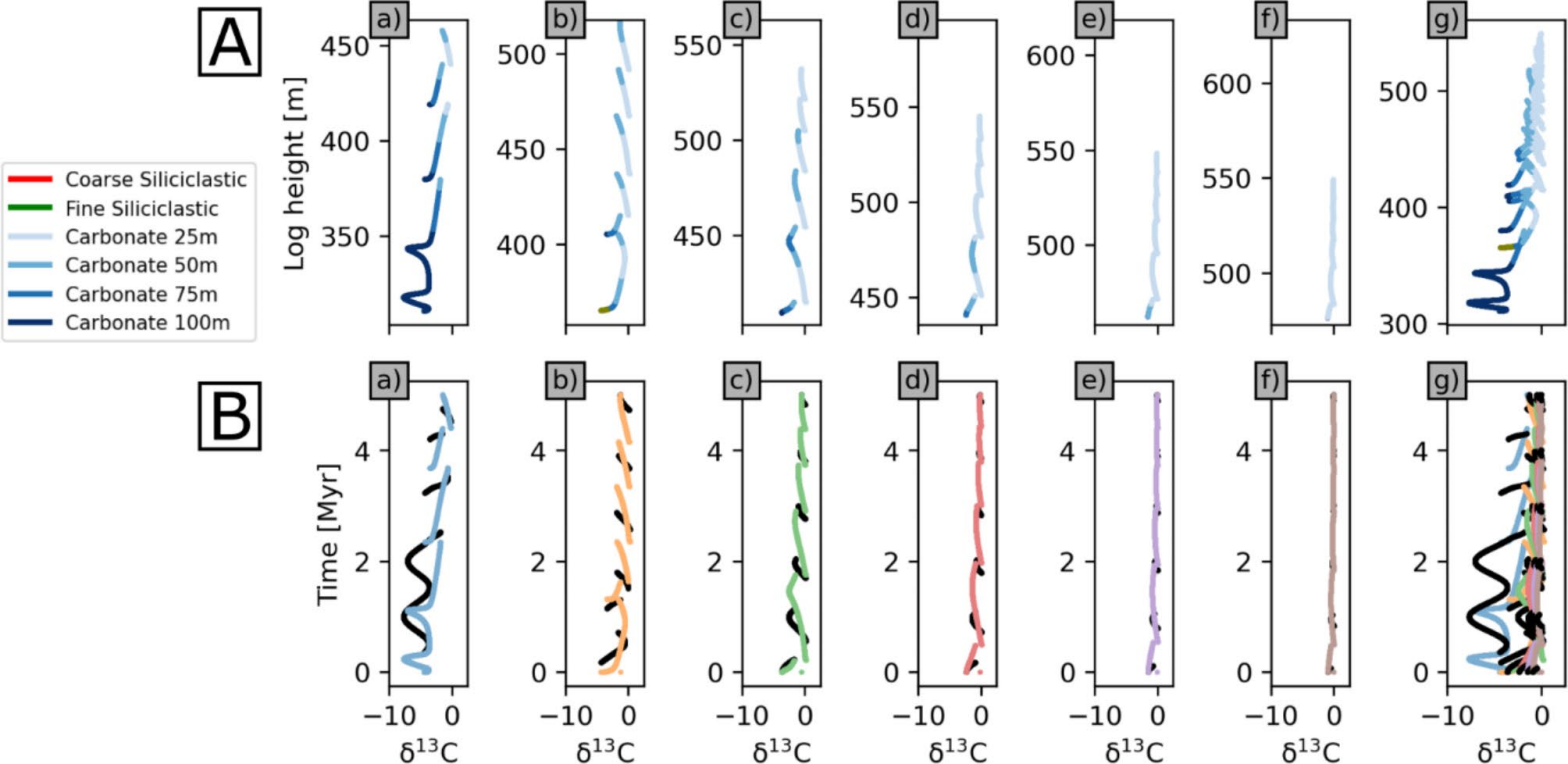
δ^13^C values in overall rising sea-level scenario. (**A**) Derived from six records (a to f; colour coded as in Fig. 5), and all combined (g), plotted by height in a 3D sedimentary succession simulated by geological process model SedSimple over 5 Myr with five 1 Myr cycles, assuming δ^13^C gradient shown in Fig. 5. (**B**) δ^13^C values in A linearly interpolated to time assuming that recorded data span the complete 5 Myr interval, with correct temporal record of preserved sediments shown in black. Note that line colours in A are mixtures of facies colours in the legend, proportionate to their concentrations. Carbonate facies are discriminated based on the depth range of deposition (25 m: up to 25 m; 50 m: 25–50 m; 75 m: 50–75 m; 100 m: 75–100 m). Line colours in B correspond to the vertical record number in Fig. 5. Vertical scales in A differ, but all records are compared at the same scale in the right-most panel.

The distribution of vertical lithologies can be combined with their modelled δ^13^C values for each record, in overall rising (Fig. 6), and falling (Figure S4) sea-level models. These are displayed both as vertical records in space as might be derived from core or outcrop height (Fig. 6A, S4A) and after interpolation to time (Ma: Fig. 6B, S4A) (See Methods, sampling records).

Even with secular variations in environmental conditions excluded, modelled vertical records show highly variable signatures that track relative water depth changes, with many step-like offsets due to hiatuses. Combined data on a single scale of vertical elevation show that offsets are highly variable in elevation (Fig. 6A(g), S4A(g)). Figures 6B, S4B compare the correct temporal record of δ^13^C values of preserved sediments (shown in black) to the spatial records in panels A after linear interpolation to time assuming that the recorded data spans the complete 5 Myr interval (shown in colours). The fragmentary and condensed nature of the record is clear in all records, but is most striking in the shallowest realms. These characteristics of the temporal record are highlighted when data from all records are combined (Figs. 6B(g), S4B(g)).

It is notable that the magnitude of δ^13^C variability is far greater in deeper records, approximately six times greater in panels (a) than panels (f) in Figs. 6 and S4. The deeper records are also more complete. This is because shallower records sample only short sections of each cycle, and these sections span similar ranges of cycle phase and hence water depth (Fig. 5). Since water depth controls δ^13^C variability, sampling the same depth in each cycle results in a relatively homogeneous signature along the shallow water record.

It is not possible to interpolate the spatial records to the correct temporal records by linear stretching unless variable hiatuses are inserted across every data offset. However, even with our continuous (infinitely finely sampled) vertical records, it is hard to identify the position of those data offsets in shallower records. This highlights a widespread issue which is likely compounded when considering discretely sampled spatial data points subject to various sources of experimental errors, and inter-regional records used to produce global temporal correlations. Given discretely sampled and noisy field data it is therefore effectively impossible to perform correct interpolation to time on individual records without additional constraints on hiatuses.

### Secular geochemical changes, with no gradient with respect to water depth

An important consequence of our results is that due to the large intervals spanned by hiatuses (Fig. 4), there is a greater than 50% probability that landward records will not preserve any short-lived event, such as the geochemical consequences of a large volcanic eruption, at all. In our models this only follows if you assume that subaerial deposition is not possible, which may not always be the case. Nevertheless, it underlines the possibility that very short-lived geochemical phenomena are not likely to provide robust signals to correlate between such records.

We now consider three frequencies of continuous secular change in seawater composition, of one, two and four cycles in 5 Myr, with an amplitude of 8‰ with no gradient with water depth. Simultaneously, sea level varies as in previous simulations.

#### Rising sea-level

**Fig. 7:**
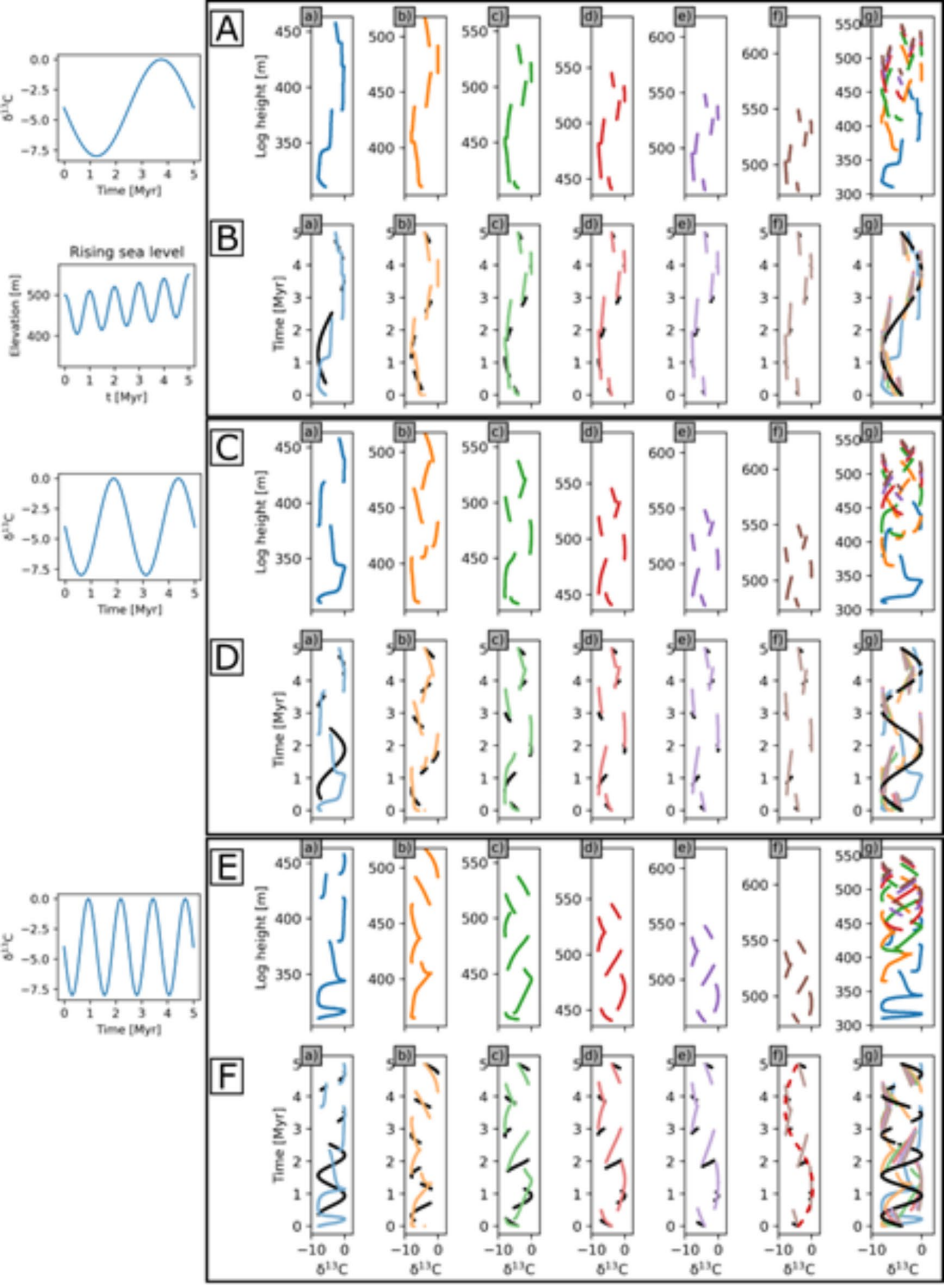
δ^13^C values and facies distribution assuming no δ^13^C gradient but three different secular changes of δ^13^C. Data derived from the six locations (a to f), and all combined (g) by height through 3D sedimentary succession simulated by geological process model SedSimple over 5 Myr with an overall rising sea-level and five 1 Myr cycles (shown on left of row **B**). Displayed by depth (**A, C, E**); time (**B, D, F**). Correct temporal record of preserved sediments shown in black, and in other colours the model spatial output linearly interpolated to time assuming that the recorded data spans the complete 5 Myr interval. Panel **F**(f) shows apparent cyclicity due to aliasing (dashed red curve). Other line colours correspond to the six locations in Fig. 5.

Figure 7 shows records in height, and the same data interpolated to geological time across the six records for an overall rising sea level. Similar to the case with geochemical gradients with respect to seawater depth, basinward records record substantially more of the temporal record than landward records, and multiple hiatuses result in large data offsets in all records. Sampling of time is so sparse in shallow water successions that a clear aliasing effect occurs: in Fig. 7F panels (b) to (f), four true secular oscillations result in a single, lower frequency apparent oscillation in the temporal record (dashed red line, panel (f)).

#### Falling sea-level

For the case of overall falling sea level the record is far less well-sampled than for the rising sea level case (Fig. S5). The most continuous records are again basinward, yet even there the true secular change is variably condensed and expanded in space or time so as to make it extremely difficult to recognise the true secular cyclicity, even for the lowest frequency case (Fig. S5B). If data were sampled at regular intervals as is currently standard in the field, many data in these records would likely be dismissed as scatter.

### Aliasing and Nyquist’s theorem

An aliasing effect occurs because temporal sampling along the records in shallow successions violates Nyquist’s theorem^34^. Whenever sampling is less dense than the Nyquist frequency (two samples per signal oscillation), the true oscillation (e.g., Fig. 7F) is irreversibly expressed as a signal with a lower *apparent* frequency (the red dashed curve in panel (f) of Fig. 7F). Since in shallow records only one short section of each cycle is recorded rather than two (Fig. 5, panel (b)), there is what we might call a *natural* sampling interval (the reciprocal of the corresponding natural sampling frequency) approximately equal to the period of sea level oscillations. Geochemical data therefore contravene the Nyquist criterion.

If the sequence (sea level) oscillation period is *τ* then its frequency is *f*_*τ*_ = 1/*τ*. We effectively obtain one sample per oscillation in Fig. 5, so the Nyquist frequency is *f*_*N*_ = 1/(2*τ*). Say the true secular oscillation in a geochemical signal has period *T* (frequency *f*_*T*_ = 1/*T*). Given the samples in Fig. 5, Nyquist’s theorem states that the true frequency *f*_*T*_ appears instead to have the apparent frequency *f*_*A*_ = |2*n f*_*N*_ - *f*_*T*_|, where *n* takes whatever integer value gives a result that lies within the range -*f*_*N*_ to + *f*_*N*_. This means that *all* true secular variations at frequencies higher than *f*_*N*_ in the geochemical data are projected into significantly lower apparent frequencies. In Fig. 7F(f), sea level oscillates with period 1 Myr so the Nyquist frequency is ½ cycles/Myr, seawater geochemistry oscillates with period 1¼ Myr so *f*_*T*_ = 4/5 cycles/Myr, and Nyquist’s formula with *n* = 1 predicts an apparent frequency *f*_*A*_ = 1/5 cycles per Myr (or one cycle per 5 Myr), which is exactly that observed in Fig. 7F(f).

A further example can be seen in Fig. 6B(f): in this case relative sea level controls both the sampling rate at 1 cycles/Myr as above, and the geochemical signal since the latter is determined by water depth. Therefore *f*_*T*_ = *f*_*τ*_ = 1 cycles/Myr and the Nyquist frequency is again ½ cycles/Myr. Nyquist’s theorem with *n* = 1 then predicts that the geochemical signal will be aliased to 0 cycles/Myr – a constant signal: this is exactly observed in Fig. 6B(f).

These two examples illustrate how Nyquist’s theorem can be applied to predict the appearance of geochemical (and equivalently, any quantitative environmental signal) in temporally under-sampled sedimentary successions. The similarity in shape between the signatures observed in rows B and F in Fig. 7 (albeit with a phase change) is therefore a result of aliasing. If a true secular change similar to that in row B occurred in one interval of time, and that in row F occurred in another, observed geochemical signatures would appear to be similar such that these two intervals might be correlated erroneously. For any records other than the two most basinward, it is therefore extremely difficult to interpret the true secular oscillation period of 1.25 Myr in Fig. 7F, even given a perfect (spatially continuous) record of data along each record. The Nyquist sampling violation is likewise visible in the falling sea-level scenario (Fig. S5F), particularly in panels (c), (d), (e) and (f) in row F, which appear to embody at most 3, 2½, 1 and 1 oscillations, respectively.

## Discussion

We use a simple model simulation to quantify where in space and time standard geochemical (or palaeontological) sampling techniques might fail to sample adequately, and use it to demonstrate how they could be improved. Even in the simple relative sea level scenarios with no secular geochemical change modelled here (which assume a dominant sea-level and water depth control on deposition), sampling deficiencies lead directly to false temporal correlations between locations (see Fig. 7F(f)). The effects of relative sea level variations (which include both the effect of absolute sea level change and subsidence) combined with either chemical gradients with depth, or secular chemical variations, are also shown to impact signatures and interpretations of standard geochemical records under apparently reasonable assumptions of past environmental conditions. Nevertheless, some scatter in data from marine basin environments may be understood systematically by deploying sequence stratigraphic concepts, such as interpretation with respect to the bounding surfaces of maximum flooding and sequences. One implication is that current methods of both sampling procedures, and temporal interpretation of geochemical records, may require modification.

Our results support conclusions from previous studies^7,24^ that the main factor challenging the correlation of disparate records is the amount of time missing at unconformities. In our examples, hiatuses span from 50 to 95% of geological time on all but the seaward-most record, and the fragmentary and condensed nature of the record is clear in any vertical records, but is most striking in the shallowest realms. Regular geochemical sampling along any vertical section therefore samples geological time highly irregularly, with each well sampled period potentially followed by a long hiatus; this pattern may be repeated in each cycle. These modelling results, even as presented in this simple scenario, demonstrate that the fragmentary and under-sampled nature of these records may potentially pose a challenge for intra-basinal and global temporal correlations: while short-lived events may be recorded in terrestrial successions that develop in rapidly subsiding basis with high rates of sediment supply, there may be a high probability that landward marine records will not preserve such events, preventing correlation between these records. The extent of the outlined biases can only truly be determined by a close examination of each published geochemical dataset within their unique stratigraphic contexts.

**Fig. 8:**
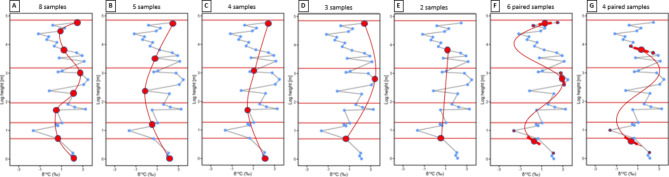
δ^13^C values (blue points, joined by thin black lines) from a sub-vertically sampled, mixed carbonate-siliciclastic ramp succession at Omkyk, Ediacaran Nama Group (Omkyk Member), Namibia, sampled in every distinct limestone bed at irregularly spaced intervals and showing the position of five high-frequency cycle boundaries (horizontal lines). Data points that would have been sampled adopting a field sampling interval of approximately 0.7 m (**A**), 1.2 m (**B**), 1.6 m (**C**), 2 m (**D**) and 2.9 m (**E**) intervals are highlighted as large red circles and joined by red lines. (**F**) and (**G**) show proposed paired-sampling (small red dots) practice to estimate average values (large red circles) and gradients (thick red lines) and a best-fitting spline function (red curve) using (**F**) six paired samples, and (**G**) four paired samples.

The effects of under-sampling and aliasing are clearly observed in Fig. 8. This shows δ^13^C_carb_ values from a high resolution, sub-vertically sampled transect through an Ediacaran mixed carbonate-clastic ramp succession (Omkyk Member, Nama Group, Namibia), spanning five high-frequency cycles, where every distinct lithological bed was sampled (significantly more dense sampling than is usual in the field). While complex, highly variable patterns are observed in the full data set (small blue circles), a clear overall signature consisting of almost two full oscillations with a wavelength of around 3 m is observed, which is resolvable using samples separated by any interval smaller than around 0.7 m (e.g., red line, panel A). This is replaced by a spurious signature consisting of a single oscillation with an apparent wavelength > 5 m if the inter-sample interval is reduced to 1.2–1.6 m (panels B to C), similarly to the aliasing example discussed above. If the inter-sample interval is reduced even further (panels D and E) only a partial cycle is observed as the true signal has been aliased to even longer wavelengths.

### Detecting and estimating aliasing effects in proxy data

Aliasing is likely to occur in many datasets consisting of proxy values sampled along individual records. Oscillatory signals with geographically and temporally varying amplitudes are often caused by Milankovitch cycles^37^. These occur on a variety of frequencies, some of which oscillate significantly more rapidly than the Nyquist frequency of field data sets and so will be aliased. In addition, any process that repeatedly perturbs an equilibrium state of a model and then returns to equilibrium may create significant oscillatory components in proxy data that again operate on a variety of timescales which may exceed the Nyquist frequency^38^.

From a theoretical point of view, there are a number of ways in which aliasing of time series can be reduced: (i) sampling regularly in time at a higher density of points increases the Nyquist frequency so that higher frequencies can be observed unaliased; (ii) applying an anti-alias filter, which removes the higher frequencies from the data, prior to or during the measurement of proxy data values; or (iii) determining the first *n* orders of temporal derivatives (gradients) of the signature of interest at each point, in addition to the signal value itself, multiplies the Nyquist frequency by *n* + 1, explained in more detail below.

In principle, sampling more densely in space along records resolves time in the rock record at higher spatial resolution. However, this provides no extra data at times of hiatus so it is not possible to sample *regularly* more closely than the natural sampling interval. Thus, strategy (i) fails.

Strategy (ii) involves removing aliased frequencies from proxy data prior to or during sampling or measurement. One possibility is to use samples or proxies for which Earth processes have already damped or removed the signatures of all dynamics that act at frequencies higher than half of the natural sampling frequency (i.e., than the Nyquist frequency). For example, say the inter-sample interval is equal to the residence time of the proxy: assuming that the proxy already averages-out variations due to processes that occur on shorter timescales (higher frequencies), aliasing effects would be removed. Since it is not possible to sample both regularly and more densely than the natural sampling interval *τ*, we require the proxy residence time *T*_*p*_ to be at least as long as the natural sampling interval. Any dynamic process that controls proxy signatures on timescales shorter than the natural sampling interval, yet longer than the proxy residence time, remain aliased.

In principle, strategy (iii) can be tested: instead of one sample at each location, two or more very closely-spaced samples can be taken, separated by a spatial interval *dz* corresponding to time interval *dt* that is sufficiently small that geological dynamics have not changed between samples. The first order temporal derivative is approximated by a finite-difference formula, *p*′(*t* + *dt*/2) = [*p*(*t* + *dt*) – *p*(*t*)]/*dt* where *p*(*t*) is the proxy value at time *t*, and if a cluster of more than two samples are taken at regular intervals *dt* then higher-order derivatives are estimated by applying this formula recursively (e.g., second-order derivatives (curvature) are approximately *p*″(*t* + *dt*/2) = [*p*′(*t* + *dt*) – *p*′(*t*)]/*dt*. However, estimating *dt* from *dx* is our ultimate goal, rather than being known a priori. By the law of superposition *dt* > 0 so the sign (+ or -) of derivatives will be observed correctly, but their magnitude scales with 1/(*dt*)^*n*^ where *n* is the order of temporal derivatives, which can only be estimated. Errors may be compounded by consideration that *dt* must be greater than the residence time of the geochemical or other proxy being explored, otherwise signatures of sample pairs are not independent. And finally, the absolute time *t* for each sample cluster remains unknown a priori due to the presence of hiatuses between sample groups.

Nevertheless, strategy (iii) implies that aliasing might be *detected* in measured data using derivatives. The temporal derivatives of signatures (slope of black line segments) are observable in Fig. 7 and S5. Using only the sign of each gradient (observable from closely spaced samples) it is clear that some indicate a local slope that goes counter to the slope of the apparent (aliased) oscillation – compare the slope of each black line segment with the corresponding slope of the dashed red curve in Fig. 7F, panel f. This indicates that the data include a higher frequency signal with peaks or troughs between each pair of sample points, and therefore that aliasing has occurred.

We illustrate with our real data set that aliasing can be detected, and the correct cyclicity can be recovered, using derivatives. In Fig. 8F each sample in Fig. 8D is replaced by the two closely spaced samples above and below (small red points), which are used to calculate an average value (large red circle) and a first order derivative or slope (thick red line). The slope at top and bottom points in Fig. 8F have the opposite sign to that of the red curve in Fig. 8D, correctly indicating that Fig. 8D is aliased. Fitting both the average and gradient values at each point using a best-fitting spline function produces the red curve in Fig. 8F, which has approximately the same cycle length as in Fig. 8A. A similar signal is constructed using only two sets of paired samples in Fig. 8G.

Aliased frequencies can be further constrained using a variant of strategy (ii). The highest possible frequency of signal resolvable by the proxy is *f*_*p*_ = 1/*T*_*p*_, since the proxy would average higher frequency oscillations approximately to zero. The aliased signal must therefore lie in the range [*f*_*N*_, *f*_*p*_]. Estimating the geological age of each sample is then a non-unique inverse problem to be solved, e.g., using computational methods such as in^11,39^, but even only the signs of spatial (and hence, temporal) derivatives of proxy data place additional constraints on those ages.

Nyquist’s theorem implies that aliased frequencies depend only on the frequencies of secular and sea level variations; estimates of these quantities allow aliased frequencies to be predicted. For example, in Fig. S5 we might first assume (correctly in this case) that sedimentologically-observed hiatuses would indicate 5 sea level oscillations between ages 0 Ma and 5 Ma, so on average *τ* = 1 Ma and *f*_*τ*_ = 1Ma^− 1^. Second, we might assume that signs of derivatives (the slopes of roughly linear segments) observed on the deepest records indicate all preserved secular oscillations, in this case 4 oscillations, giving an average *Τ* = 1.25 Ma and frequency *f*_*Τ*_ = 0.8. Identically to the calculation above, Nyquist’s theorem then correctly predicts an apparent frequency *f*_*A*_ = 1/5Ma^− 1^ on the shallow record.

Thus, we correctly predict how the true geochemical secular frequency observed on the basinward record will be expressed on the shallowest record. Careful field observations would then allow the low-frequency aliased signal (Fig. 7F(f)) to be correlated correctly with the higher-frequency unaliased signal (Fig. 7F(a)).

Uncertainties in δ^13^C measurements are normally estimated in the range 0.08‰ to 0.1‰, which has little effect on the slope of derivatives in our examples, and would change the sign of none leaving the discussion above intact. However, these estimates are purely analytical, so do not account for fluctuations due to sampling different grain compositions in laterally heterogeneous rock mass. It is therefore unproductive to analyse the propagation of such under-estimates other than in specific examples.

A more general approach to understand error propagation is to consider the effect of using derivative data in the frequency domain. If the geochemical proxy signal is *p*(*t*) then (using capitals for Fourier domain quantities) the transform of this function is *P*(*f*) for frequency *f*. Our *n* data samples are then {*d*(*t*_*i*_): for times *t*_1_,…,*t*_*n*_}, and each sample contains an error which we denote *e*(*t*_*i*_), so *d*(*t*_*i*_) = *p*(*t*_*i*_) + *e*(*t*_*i*_). The Fourier transform is linear, so taking the discrete Fourier transform of our data gives *D*(*f*) = *P*(*f*) + *E*(*f*), and from standard Fourier theory the temporal derivatives are given by *D*′(*f*) = *f P*(*f*) + *f E*(*f*). Thus, if true errors in the geochemical data are *E*(*f*) when expressed in the Fourier domain, they are amplified by a factor *f* after taking derivatives; therefore, higher frequency errors will be amplified relative to those at lower frequencies in derivative data. Further errors are incurred by using finite-difference approximations of derivatives, as discussed above.

While this sensitivity to errors is undesirable, it is in principle more than compensated by the fact that if rock from a certain time period is missing from the record, then it cannot be sampled. Gradient measurements allow similar information, albeit with increased uncertainty, to be obtained from neighbouring samples as demonstrated by comparing panels A and F in Fig. 8.

Finally, it is possible to estimate full uncertainties on correlations, and to predict (image tomographically) stratigraphic and geochemical structures that occur between geographical locations of even aliased geochemical data, by introducing geological process information to the correlation directly. Conceptual and dynamic information in geological process models calibrated to real-life examples, i.e., to each unique depositional scenario, can be embodied within a machine learning architecture as in^12,40^. These studies show that a computational method can then be established to fit geophysical or geochemical data to within their observational uncertainties. If the process model is sufficiently sophisticated to represent geological processes that control the data in question, and provided that sufficient computational power is available to represent the family of possible three-dimensional structures that might arise, then this computational approach characterises the range of possible correlations. With aliased samples those correlation uncertainties may increaserelative to unaliased samples, but nevertheless, in principle this method allows those uncertainties to be reduced by the introduction of process information, and quantified.

## Methods

We use a geological process model (GPM) called *SedSimple*^41^ to generate exemplar mixed carbonate-siliciclastic shelf margin sedimentary sequences. A GPM is a numerical simulation of dynamic processes acting over geological timescales^41–44^. The range of processes included depends on the environment to be modelled, but for sedimentary environments typically includes most of sedimentary deposition, carbonate growth, erosion, transportation, re-deposition, and tectonic processes^45^. Given a base topography, relative sea level as a function of time, and rate of carbonate production as a function of water depth, the GPM simulates sedimentary siliciclastic deposition and carbonate production, plus erosion and redistribution of both carbonate and siliciclastic sediments, resulting in a three-dimensional sedimentary volume. *SedSimple* is designed for rapid execution while simulating all of the above processes (see Supplementary Material).

We simulate 5 Myr during which we form five sequences or parasequences (henceforth, sequences) due to relative sea level oscillations of 1 Myr duration, and compare a scenario of overall (time-averaged) rising sea-level with one of overall falling sea-level across an initial topography of a platform to basin profile, with carbonate growth rate a function of water depth (Figure S1). We refer to relative sea level simply as sea level – the effects of subsidence are implicit. Our simulation includes deposition, carbonate growth, erosion, sedimentary diffusion and re-deposition. We model three discrete facies which become mixed during diffusion: two siliciclastic facies of different grain sizes (fine and coarse) and one carbonate facies deposited at different water depths segmented into four depth ranges (25 m: 0 to 25 m depth; 50 m: 25 to 50 m depth; 75 m: 50 to 75 m depth; 100 m: 75 to 100 m depth).

The simulated model contains the proportion of each facies at every 3D location and time step. It provides information on where and when each facies was deposited, which in combination with sea level variations allows water depth at each location and time of deposition to be inferred throughout the model. We further model the δ^13^C values recorded in sediment as linearly decreasing with depositional water depth, values also available throughout the model.

We finely sample six vertical records spaced regularly between basinward and landward positions, simulating ultra-dense sub-vertical geochemical sampling for each location. These records are then interpolated to time, assuming the uncommonly fortunate scenario that a dated horizon such as an ash bed is available at the bottom (0 Ma) and top (5 Ma) of the succession – time values increase through the simulation. Data for each location are interpolated linearly between these ages to create temporal records, as is common practice. The resulting records represent idealised representations of geochemical data sets, from multiple, well-distributed records, with essentially continuous vertical spatial sampling. This represents far denser sampling than is currently undertaken in practice.

Many examples are known of major δ^13^C gradients with water depth, such as the modern Baltic Sea^46^, the Ediacaran Wumishan Formation^47^ and Ediacaran successions^33^ of south China. Here we use a Cryogenian case study which demonstrates differences in δ^13^C of between 8 and 11‰ from back-reef to basin as an example gradient of stratification of δ^13^C with water depth^36^. While our chosen value of 10‰ might appear to be on the high end of gradient values, scaling the gradient by any factor (e.g., 0.5 for a desired gradient of 5‰) simply scales our simulated geochemical values by the same factor. We record resultant modelled δ^13^C values in the deposited carbonate sediments (not siliciclastics) only.

## Electronic supplementary material

Below is the link to the electronic supplementary material.

Supplementary Material 1

## Data Availability

All new data are available in the supplementary material.
