# Supplementary material for: Natural sampling and aliasing of marine geochemical signals
Source: Sci Rep. 2025 Jan 4;15:760. doi: 10.1038/s41598-024-84871-6 (PMC11700088; doi:10.1038/s41598-024-84871-6)

Supplementary Information

Natural sampling and aliasing of shallow-marine environmental signals

Andrew Curtis, Hugo Bloem, Rachel Wood, Fred Bowyer, Graham A. Shields, Ying Zhou, Mariana Yilales, Daniel Tetzlaff

Supplementary Text
Figures S1-5

Supplementary Text

SedSimple

SedSimple by Tetzlaff (2023) is open-source software and free for academic use and is available here: https://wsoftc.com/index.php/software. SedSimple offers different geological processes: sedimentation, erosion, transport, channel modelling, wave action, carbonates, aggradation, and tectonics. The following provides an overview of a selection of these processes as used in this work, a more comprehensive review can be found in Tetzlaff and Harbaugh (1989) and Tetzlaff (2023).

Sedimentation, erosion and transport

Consider two domains: one above the topographic surface, which can be either the water column or, in the absence of water, the atmosphere, and the other where sediments accumulate and rocks form. These sediments can be categorized into S facies types, each with representative properties. Erosion or deposition of sediments can be viewed as an exchange

of matter between the two domains and can be mathematically described using the continuity equation (Tetzlaff and Harbaugh, 1989):

$$(H - Z)\sum_{s=1}^{S}\frac{dK_s}{dt} = \frac{\partial Z}{\partial t}$$

where, H represents the free-surface elevation, Z is the topographic elevation, K_s stands for the volumetric sediment concentration of the sth sediment type, and t denotes time. A change in sediment at a specific location over time, $\frac{dK_s}{dt}$, influences the height of the topographic surface Z. Once entrained, sediments can be transported by various phenomena, primarily bed load and suspended load transport. Bed load transport involves sediment being rolled over the bed due to the force of flowing liquid, while suspended load transport relates to the movement of sediment when it is suspended in the fluid (Chien and Wan, 1999). However, SedSimple does not explicitly model these processes. In largescale simulations, such as stratigraphic forward modelling, the focus is on the amount of sediment transported rather than the specific transport mechanisms. Consequently, SedSimple simulates sediment flux, representing the volume of sediment passing through a specific plane over time.

The calculated sediment flux also computes sediment diffusion, which describes the movement of particles from areas of high concentration to low concentration. In sedimentary systems, this can occur either from a topographic high to a low or from regions of high to low suspended concentration. Both are governed by the same diffusion equation:

$$\frac{\partial z}{\partial t} = k\nabla^2 z + s_s$$

Here, k represents the diffusion coefficient, ∇^2 is the Laplacian operator, and s_s is the sediment source term. The value of the diffusion coefficient k depends on both the sediment's transportability and the local environment. For instance, areas with high energy,

like coastlines with breaking waves, have larger diffusion coefficients compared to low-flow regions such as the deep sea (Kaufman et al., 1991).

Different sediments respond differently to the processes described above due to factors like particle diameter and density. Sediment particles with distinct properties may erode and transport more easily. Rather than modelling these properties separately, SedSimple employs a unified concept known as the transportability coefficient (Tetzlaff, 2023). This dimensionless property quantifies the ease with which a particle can be transported, either by flow or by gravity such as in diffusion, and is derived from the particle's fall velocity.

Carbonates and Aggradation

Carbonate grow rate depends on the nutrients available and light levels due to water depth (Demicco and Hardie, 2002). The depth dependency can be represented by a function that relates relative growth rate and water depth. Multiplying this by a maximum production value (mm/a) yields the carbonate growth rate. SedSimple uses this approximation with any desired growth curve or production value. A similar approach can be taken for aggradation in deep-sea sedimentation. However, deep-sea sedimentation of for example foraminifera growing in the water column, has an opposite relation to water depth compared to carbonate production, up to water depths where enough light is available. This can be modelled by introducing a different relative growth function over depth and an appropriate maximum production value to simulate this behaviour (Tetzlaff, 2023).

Figure S1. Model parameters used in the geological process model SedSimple over 5 Myr with five 1 Myr cycles. A, overall rising; B, overall falling sea-level; C) initial topography of shelf to basin; D) modelled carbonate growth (production) rate multiplier.

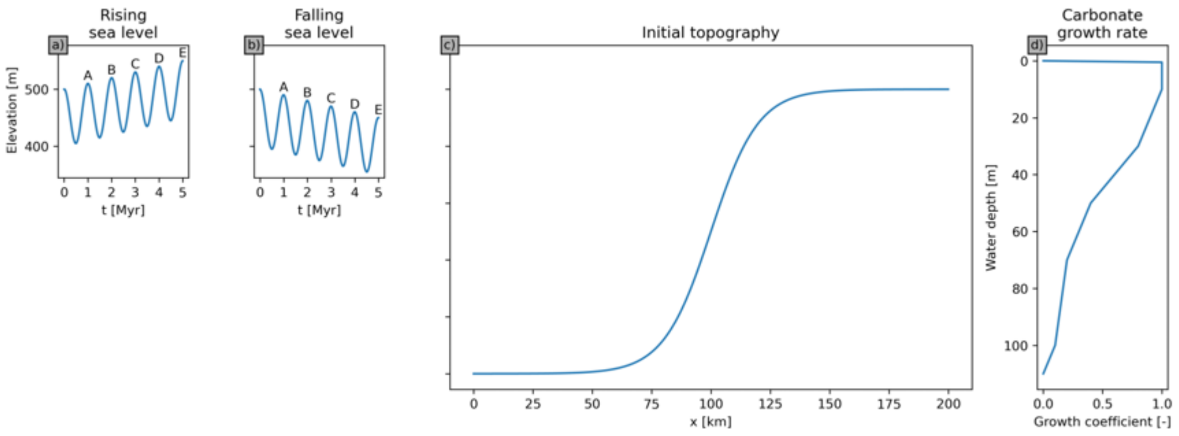

Figure S2: Cross-sections and vertical records through 3D sedimentary succession simulated by geological process model SedSimple with 1 Myr sea level cycles with an overall falling sea-level. A: location of six vertical records (1-6) over 5 Myr to show distribution of a) timing of deposition. b) facies deposited, with dotted horizontal sections of curves show hiatuses. c) total percentage of time missing (hiatuses) in each record. B: detail with a) vertical exaggeration of records over first 2 Myr showing the distribution of sample locations on each vertical record (small triangles) that represent regular sampling of geological time on the horizontal axis. b) facies deposited, with dotted horizontal sections of curves show hiatuses. Regularly spaced geological time samples (red dots between 0.25 and 1 Ma) are projected (arrows) to elevations on each record at which those times are recorded in the sedimentary record. Dashed arrows show time samples preserved on more than one record.

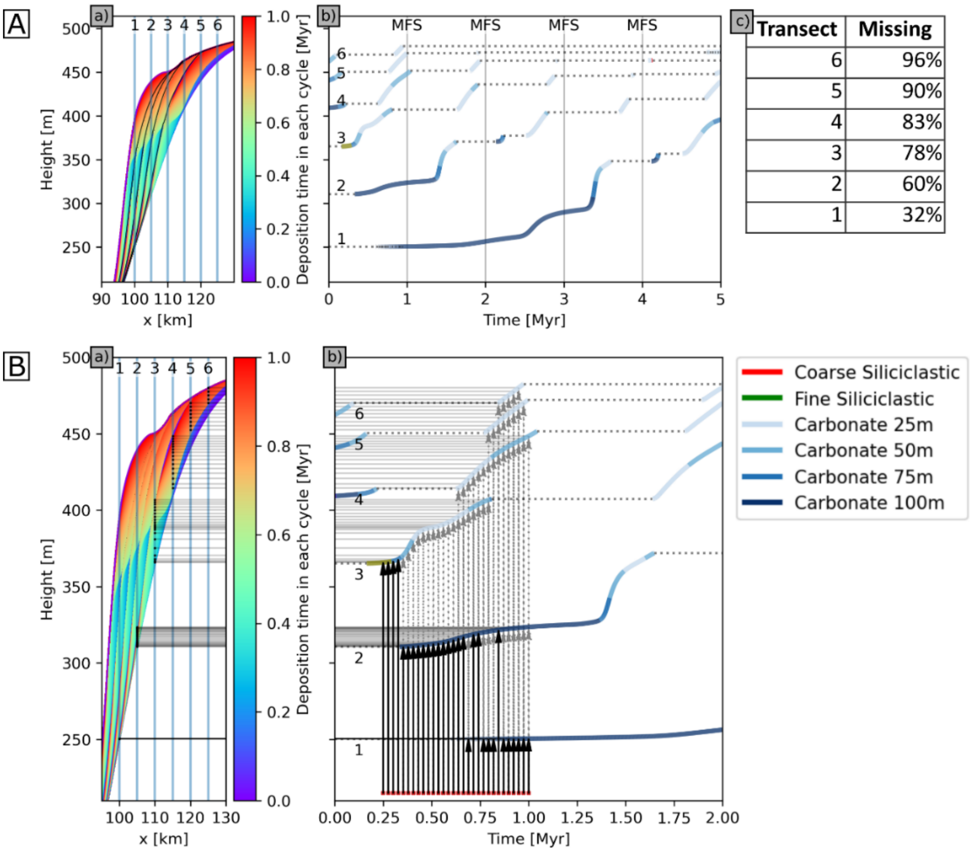

Figure S3: Sections through 3D sedimentary succession showing water depth changes simulated by geological process model SedSimple over 5 Myr with five 1 Myr sea level cycles during overall falling sea-level. a) Water depth of deposition within each 1 Myr cycle, with locations of six vertical records (colour coded). (b) Water depth and $\delta^{13}C$ values in each (colour coded) record through time, assuming gradient shown on left vertical axis. Grey lines show full water depth history along each record. Black dots show values at the regularly spaced time intervals between 0.25 and 1 shown in Fig. 4B(b). MFS = Maximum Flooding Surface.

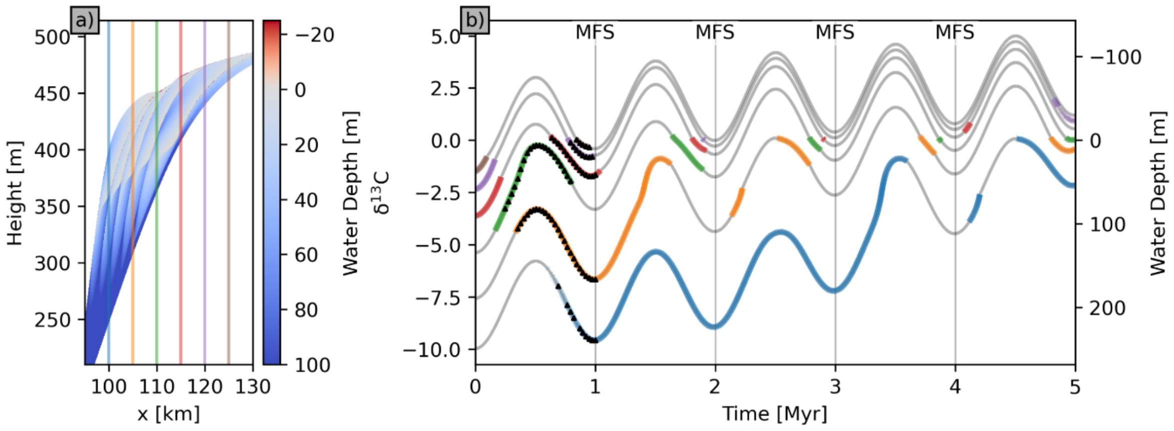

Figure S4: δ^{13}C values in overall falling sea-level scenario. A: Derived from six records (a to f; colour coded as in Fig. 5), and all combined (g), plotted by height in a 3D sedimentary succession simulated by geological process model SedSimple over 5 Myr with five 1 Myr cycles, assuming δ^{13}C gradient shown in Fig. 5. B: δ^{13}C values in A linearly interpolated to time assuming that recorded data span the complete 5 Myr interval, with correct temporal record of preserved sediments shown in black. Note that line colours in A are mixtures of facies colours in the legend, proportionate to their concentrations. Carbonate facies are discriminated based on the depth range of deposition (25m: up to 25m; 50m: 25-50m; 75m: 50-75m; 100m: 75-100m). Line colours in B correspond to the vertical record number in Fig.

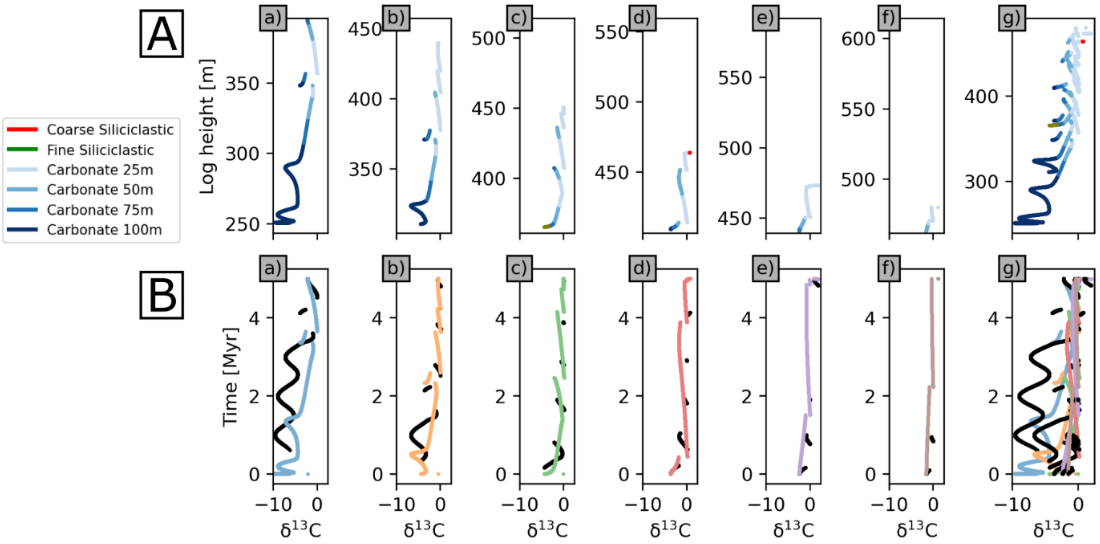

Figure S5: δ^{13}C values and facies distribution assuming no δ ^{13}C gradient but three different secular changes of δ^{13}C with an overall falling sea-level. Data derived from the six locations (a to f), and all combined (g) by depth through 3D sedimentary succession simulated by geological process model SedSimple over 5 Myr with an overall falling sea-level and five 1 Myr cycles by A,C,E: depth; B,D,F: in time. Correct temporal record of preserved sediments are shown in black, and other colours represent the model spatial output (A,C,E) linearly interpolated to time assuming that the recorded data spans the complete 5 Myr interval. Panel F(f) shows apparent cyclicity due to aliasing (dashed red). Other line colours correspond to the six locations in Fig. 5.

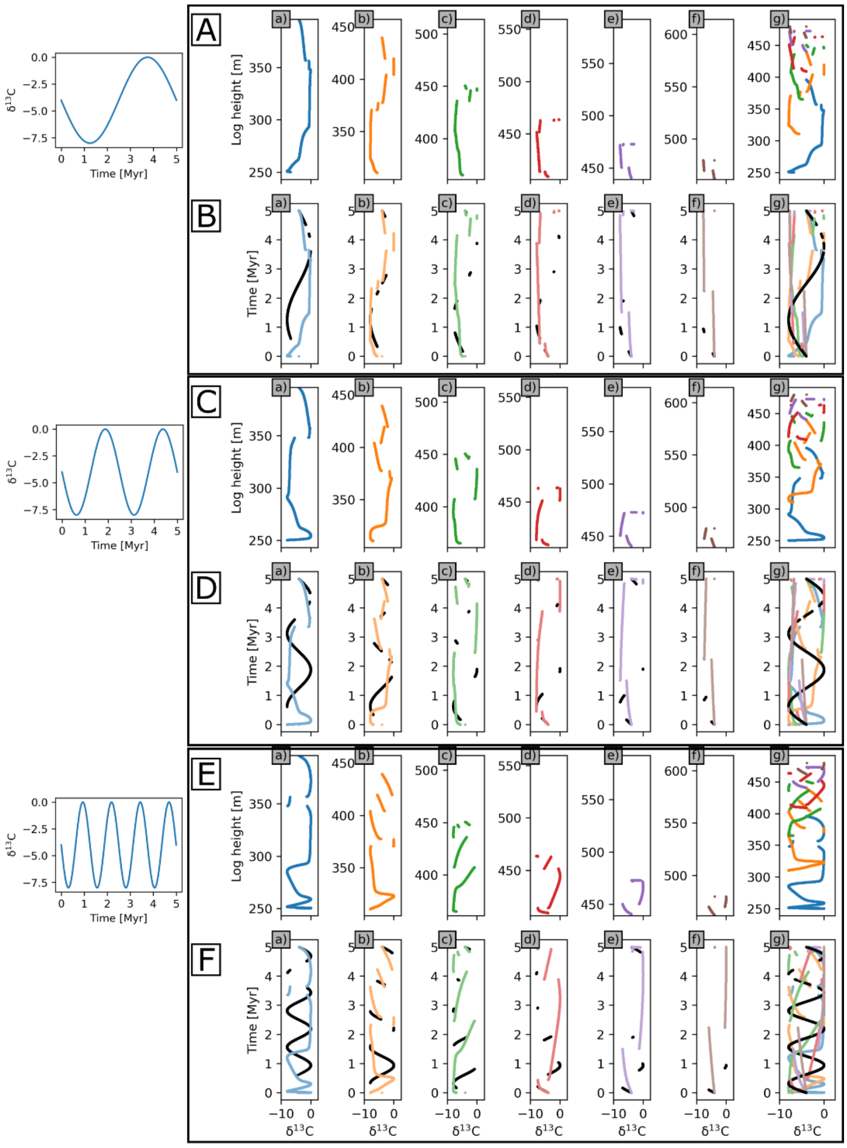

Supplement: Supplementary file 1 — Supplementary Material 1 [file 41598_2024_84871_MOESM1_ESM.pdf]
